# Supplementary material for: Potential drivers for schistosomiasis persistence: Population genetic analyses from a cluster-randomized urogenital schistosomiasis elimination trial across the Zanzibar islands
Source: PLoS Negl Trop Dis. 2022 Oct 10;16(10):e0010419. doi: 10.1371/journal.pntd.0010419 (PMC9584424; doi:10.1371/journal.pntd.0010419)
Supplement: S1 Text — Fig A. Adjusted mean ((± 95% confidence interval) allelic richness (Ar) by island, intervention arm and year where where Pemba MDA arm n = 41 baseline, n = 22 year 5; Snail arm n = 40 baseline, n = 55 year 5; Behaviour arm n = 29 baseline, n = 37 Year 5; and where Unjuga MDA arm n = 20 baseline, n = 18 year 5; Snail arm n = 46 baseline; n = 8 year 5; Behaviour arm n = 43 baseline, n = 31 Year 5. Figs B and C. Mean (± 95% confidence interval) (b) Expected Heterozygosity (He) and (c) Observed Heterozygocity (Ho) by island, intervention arm and year where Pemba MDA arm n = 41 baseline, n = 22 year 5; Snail arm n = 40 baseline, n = 55 year 5; Behaviour arm n = 29 baseline, n = 37 Year 5; Fig D. Mean (± 95% confidence interval) inbreeding coefficient (FST) by island, intervention arm and year by island, intervention arm and year where Pemba MDA arm n = 41 baseline, n = 22 year 5; Snail arm n = 40 baseline, n = 55 year 5; Behaviour arm n = 29 baseline, n = 37 Year 5. The inbreeding coefficient is a measure of the degree of similarity between parasites within an individual where a lower value indicates less relatedness. (DOCX) [file pntd.0010419.s002.docx]

**Supplementary Information**

***Microsatellite Genotyping.***

Miracidia were genotyped for 15 microsatellite loci using two *S. haematobium* multiplexed microsatellite PCR’s Table A). The panels the alleles were amplified in together with the allele repeat and size range.

**Table A. *Schistosoma haematobium* microsatellite PCR panels 1 and 2**

|  | **Loci** | **Forward Primer 5'- 3'** | **Reverse Primer 5'- 3'** | **Dye** | **Size Range**  **(bp)** | **Repeat** |
| --- | --- | --- | --- | --- | --- | --- |
|  |  |  |  |  |  |  |
| **Panel 1** | C102 | TGTCTCTGTGAATGACCGAAT | TTAGATGAATAATAATGTTGAAACCAC | VIC | 184-199 | ATT |
|  | Sh1 | GCATCCAATTTCGTACAC | CCACATTAGGCCAACAAG | VIC | 245-284 | AAT |
|  | Sh14 | GTCCTCCTTCCCTCTTTG | CACATTCGTCCTAGATATCG | NED | 184-240 | ACTC |
|  | C131 | CTTGTCATTTGGGCATTGTG | CATGGTGAGGTTCAAACGTG | NED | 253-265 | AAT |
|  | Sh6 | GGTGGATTACGCAATAG | TTTAATCAACCGGGTGTC | NED | 309-321 | AAT |
|  | Sh9 | GGGATGTATGCAGACTTG | TTGTTTGGCTGCAGTAAC | 6-FAM | 197-227 | AAT |
|  | Sh3 | GCTGAGCTTGAGATTG | CTTCTGTCCCATCGATACC | 6-FAM | 270-366 | AAT |
|  | C111 | CCCTTGTCTTCAATGCGTTA | GAACGTCTAACTGGCGATCA | PET | 201-225 | ATT |
|  |  |  |  |  |  |  |
| **Panel 2** | Sh2 | TTAGTGTGTTTGGCTTCAAC | CCTCGAATGAAATCCTGAC | NED | 155-218 | AAT |
|  | Sh5 | TGTGCACAAGAAAGATTAAATG | ACGACAATGTTGCAAGTTC | NED | 263-314 | AAT |
|  | Sh13 | GAGCAGCTATTTCGTATCG | ACCGTGGACAGTTCATCAG | 6-FAM | 163-211 | AAT |
|  | Sh4 | CCCATCGCTGATATTAAAG | TCTAGTCGTCTTGGGATCC | 6-FAM | 268-313 | AAT |
|  | Sh10 | CGCATGTCATACCTATCTCC | GCTTATCAGGCCTATCTCC | PET | 183-207 | AAT |
|  | Sh12 | CGTCTTAGTGAGCCAGATG | CTCGTGGACATCATCAG | PET | 245-278 | AAC |
|  | Sh15 | CTTTCAGTAGGATTTGTTG | CGACGTCAAGCACTGTAC | VIC | 274-301 | ATC |

[1-3].

**FTA DNA alkaline elution**

# Samples were handled in a 96 well format to allow high through put processing and analysis. DNA elutions were carried out in low profile 1.2 ml 96 square well storage microplates with 96 square well sealing cap mats (Fisher scientific) which facilitates DNA elution. The eluted DNA was then transferred to a suitable 96 well storage vessel and either used immediately or stored at -20**°**C for future use.

# **Method**

# Punch out 2mm FTA disc, containing the larval sample, using a Micro Harris Punch.

# Add: 14μl of Solution 1 (0.1 N NaOH, 0.3 mM EDTA, pH 13.0).

# Incubate at room temperature for 5 mins

# Add: 26μl of solution 2 (0.1 M Tris-HCl, pH 7.0) and seal the plate

# Pulse vortex 3 times

1. Incubate at room temperature for 10 mins
2. Pulse vortex 10 times
3. Remove the elute (DNA) and store frozen or use.

[1]

**Microsatellite PCR (12.5μl reaction for each Panel)**

For each batch of reactions a master mix was made containing the following reagents.

- 6.25μl 2X Type-it**^®^** Microsatellite PCR Kit (Qiagen)
- 1.25μl Q solution ((Type-It Microsatellite Kit (Qiagen))
- 1.25μl Primer mix containing 0.2 μM of each primer (refer to the primer mix protocol in the Type-it Microsatellite PCR Kit (Qiagen) handbook).
- 1.75 μl H_2_O
- 2 μl DNA elute

All reactions were set up at room temperature in 96 well PCR plates

The Microsatellite **PCR cycle consisted of** and initial denaturing step of 95°C for 5 mins followed by 32 cycles of 95°C for 30 sec, 54 °C for 1min 30sec, 72°C for 3 mins and a final elongation of 60°C for 30 mins. Positive reactions were diluted 1 in 10 with 2μl of each reaction being mixed with 0.35μl of GS500Liz size standard before being denatured for 5 mins at 95°C and injected at a 12-second injection speed into the Applied Biosystems 3130xl DNA Analyser for fragment analysis [1].

**Table B. Number of infected hosts sampled for population genetic analyses per island, age group, intervention arm and year.**

|  | Age | 2012 | Intervention A1:A2:A3 | Age | 2016 | Intervention  A1:A2:A3 |
| --- | --- | --- | --- | --- | --- | --- |
| Pemba | 6-8 | 53 | 18:18:17 | 6-8 | n.a. | n.a. |
|  | 9-12 | 31 | 11:15:5 | 9-12 | 102 | 19:50:33 |
|  | 13-75 | 26 | 12:7:7 | 20-30 | 12 | 3:5:4 |
|  |  |  |  |  |  |  |
| Unguja | 7-8 | 32 | 5:13:14 | 7-8 | n.a. | n.a. |
|  | 9-12 | 59 | 15:29:15 | 9-12 | 44 | 9:8:27 |
|  | 20-54 | 18 | 0:4:14 | 20-46 | 13 | 9:0:4 |
|  |  |  |  |  |  |  |

Where:

A1 – MDA intervention arm

A2- MDA + snail intervention arm

A3 MDA + behaviour intervention arm

n.a. not applicable as no sampling of children <9 years old was performed post baseline.,

**Table C**. **Number of miracidia randomly-selected for amplification from *n* individuals in each year, island and intervention arm.**

| Year | Island | Total (n) | MDA (n) | MDA + B (n) | MDA + S (n) |
| --- | --- | --- | --- | --- | --- |
| 2012 | Unguja | 946 (109) | 150 (20) | 392 (43) | 404 (46) |
|  | Pemba | 879 (110) | 296 (41) | 252 (29) | 331 (40) |
| 2016 | Unguja | 558 (57) | 165 (18) | 316 (31) | 77 (8) |
|  | Pemba | 1142 (114) | 227 (22) | 336 (37) | 579 (55) |

**Table D. Number of miracidia randomly selected for amplification from *n* individuals by gender.**

| Year | Gender | Total (n) |
| --- | --- | --- |
| 2012 | Female | 676 (77) |
|  | Male | 1149 (142) |
| 2016 | Female | 726 (73) |
|  | Male | 974 (98) |

**Fig A. Adjusted mean allelic richness (Ar) by island, intervention arm and year.**

**Fig B. Expected Heterozygosity (He) by island, intervention arm and year.**

**Fig C. Observed Heterozygosity (Ho) by island, intervention arm and year.**

**Fig D. Mean inbreeding coefficient (*F*_ST_) by island, intervention arm and year.**

**Figure Legends**

**Fig A.** Adjusted mean ((± 95% confidence interval) allelic richness (Ar) by island, intervention arm and year where where Pemba MDA arm n=41 baseline, n=22 year 5; Snail arm n=40 baseline, n=55 year 5; Behaviour arm n=29 baseline, n=37 Year 5; and where Unjuga MDA arm n=20 baseline, n=18 year 5; Snail arm n=46 baseline; n=8 year 5; Behaviour arm n=43 baseline, n=31 Year 5.

**Figs B and C.** Mean (± 95% confidence interval) (b) Expected Heterozygosity (He) and (c) Observed Heterozygocity (Ho) by island, intervention arm and year where Pemba MDA arm n=41 baseline, n=22 year 5; Snail arm n=40 baseline, n=55 year 5; Behaviour arm n=29 baseline, n=37 Year 5;

**Fig D.** Mean (± 95% confidence interval) inbreeding coefficient (*F*_ST_) by island, intervention arm and year by island, intervention arm and year where Pemba MDA arm n=41 baseline, n=22 year 5; Snail arm n=40 baseline, n=55 year 5; Behaviour arm n=29 baseline, n=37 Year 5. The inbreeding coefficient is a measure of the degree of similarity between parasites within an individual where a lower value indicates less relatedness.

**Additional References cited**

1. Webster, B.L., et al., *Development of novel multiplex microsatellite polymerase chain reactions to enable high-throughput population genetic studies of Schistosoma haematobium.* *Parasites & Vectors*, 2015. **8**(1): p. 432.

2. Gower, C.M., et al., *Population genetics of Schistosoma haematobium: development of novel microsatellite markers and their application to schistosomiasis control in Mali.* Parasitology, 2011. **138**: p. 978-994.

3. Glenn, T.C., et al., *Significant variance in genetic diversity among populations of Schistosoma haematobium detected using microsatellite DNA loci from a genome-wide database.* Parasites & Vectors, 2013. **6**(1): p. 300.
