# Supplementary material for: Potential drivers for schistosomiasis persistence: Population genetic analyses from a cluster-randomized urogenital schistosomiasis elimination trial across the Zanzibar islands
Source: PLoS Negl Trop Dis. 2022 Oct 10;16(10):e0010419. doi: 10.1371/journal.pntd.0010419 (PMC9584424; doi:10.1371/journal.pntd.0010419)
Supplement: Attachment — Submitted filename: Responses to referees PLoS NTDS Zanzibar_2JPW.docx [file pntd.0010419.s003.docx]

**Responses to Nithya Venkatesan (Formatting)**

*Manuscript ID: PNTD-D-22-00475R1


Thank you for submitting your paper, "Potential drivers for schistosomiasis persistence: population genetic analyses from a cluster-randomized urogenital schistosomiasis elimination trial across the Zanzibar islands", to PLOS Neglected Tropical Diseases. We have checked over your submission and, before we can assign your manuscript to the Editor, we ask that you first please make the following modifications:

1) Please upload all main figures as separate Figure files in .tif or .eps format.*

Amended – all have been uploaded as tif files.

*2) Please ensure that all author names are spelled correctly in the manuscript file, including the use of a middle name/initials, and any special characters (accents, umlauts, etc.). Please place first/given names before last/family names.  All authors must have a first and last name. We noticed that "M. Ines Neves, Iddi Khamis, Mtumweni A Muhsin, Khamis R Suleiman, Fatama Kabole, Martin Walker" are spelled differently between the manuscript file and submission form. Please be aware that correct spelling and formatting of author names in the article file is vital for accurate indexing in PubMed.*

Apologies and amended
 *3) We do not publish any copyright or trademark symbols that usually accompany proprietary names, eg (R), (C), or TM  (e.g. next to drug or reagent names). Please remove all instances of trademark/copyright symbols throughout the text, including MICROLAB® on line no 182, 185.*

Amended

*4) We have noticed that you have uploaded Supporting Information files, but you have not included a list of legends. Please add a full list of legends for your Supporting Information files after the references list.*

Amended
 *5)  Please ensure that the funders and grant numbers match between the Financial Disclosure field and the Funding Information tab in your submission form. Note that the funders must be provided in the same order in both places as well.
 Currently, the funder name "Wellcome Trust" and the grant number "RR374-053/4893206" are missing from the financial disclosure and "University of Georgia Research Foundation, Inc (UGARF)" is missing from the funding information.*

Thank you and amended

However, as regards RR374-053/5054146 & RR374-053/4785426, the online drop selection option from the funding information box does not give me the opportunity to add UGARF to either the Bill and Melinda Gates option, as is relevant here, nor allow me to add independently. Thus whilst this is correct within our document main text, I cannot add this to the dropdown box. Full details are, however, provided in the revised summary box.

*6) Thank you for stating" Coding, combined with anonymised microsatellite data associated with the representative schistosome samples, are all available on GitHub (*[*https://eur01.safelinks.protection.outlook.com/?url=https%3A%2F%2Fgithub.com%2Fmineneves%2FZanzibar_population-genetics&amp;data=05%7C01%7Cjowebster%40rvc.ac.uk%7Cfdd5a5438192439cdfdf08da7617fa6d%7C45a3be7c94024fbead438d1faebfb42d%7C0%7C0%7C637952142603394624%7CUnknown%7CTWFpbGZsb3d8eyJWIjoiMC4wLjAwMDAiLCJQIjoiV2luMzIiLCJBTiI6Ik1haWwiLCJXVCI6Mn0%3D%7C3000%7C%7C%7C&amp;sdata=ikBkXkv1vPYqodjWajkftG%2FgB0xMmXmrTKICDhQxl0I%3D&amp;reserved=0*](https://eur01.safelinks.protection.outlook.com/?url=https%3A%2F%2Fgithub.com%2Fmineneves%2FZanzibar_population-genetics&amp;data=05%7C01%7Cjowebster%40rvc.ac.uk%7Cfdd5a5438192439cdfdf08da7617fa6d%7C45a3be7c94024fbead438d1faebfb42d%7C0%7C0%7C637952142603394624%7CUnknown%7CTWFpbGZsb3d8eyJWIjoiMC4wLjAwMDAiLCJQIjoiV2luMzIiLCJBTiI6Ik1haWwiLCJXVCI6Mn0%3D%7C3000%7C%7C%7C&amp;sdata=ikBkXkv1vPYqodjWajkftG%2FgB0xMmXmrTKICDhQxl0I%3D&amp;reserved=0)*)". This link reaches a 404 page.please amend this to a new link or provide further details to locate the data.*

Apologies, this must have been a cut and paste issue and should be corrected and functional now.

[https://www.github.com/minesneves/Zanzibar_population_genetics](https://eur01.safelinks.protection.outlook.com/?url=https%3A%2F%2Fwww.github.com%2Fminesneves%2FZanzibar_population_genetics&data=05%7C01%7Cjowebster%40rvc.ac.uk%7Ccfe330cfc9da46e85cc908da77a46001%7C45a3be7c94024fbead438d1faebfb42d%7C0%7C0%7C637953845107844869%7CUnknown%7CTWFpbGZsb3d8eyJWIjoiMC4wLjAwMDAiLCJQIjoiV2luMzIiLCJBTiI6Ik1haWwiLCJXVCI6Mn0%3D%7C3000%7C%7C%7C&sdata=ZBDsSumGpaif9ADndCrW%2FpZ70Iw5vZDyohTh9l3TPZA%3D&reserved=0)

| <https://eur01.safelinks.protection.outlook.com/?url=https%3A%2F%2Fgithub.com%2Fminesneves%2FZanzibar_population_genetics&data=05%7C01%7Cjowebster%40rvc.ac.uk%7Ccfe330cfc9da46e85cc908da77a46001%7C45a3be7c94024fbead438d1faebfb42d%7C0%7C0%7C637953845107844869%7CUnknown%7CTWFpbGZsb3d8eyJWIjoiMC4wLjAwMDAiLCJQIjoiV2luMzIiLCJBTiI6Ik1haWwiLCJXVCI6Mn0%3D%7C3000%7C%7C%7C&sdata=aq5rkYEkZSsmptca52gBH5zlsp3VLHlOFr7lpR1kkBw%3D&reserved=0> | [GitHub - minesneves/Zanzibar_population_genetics](https://eur01.safelinks.protection.outlook.com/?url=https%3A%2F%2Fgithub.com%2Fminesneves%2FZanzibar_population_genetics&data=05%7C01%7Cjowebster%40rvc.ac.uk%7Ccfe330cfc9da46e85cc908da77a46001%7C45a3be7c94024fbead438d1faebfb42d%7C0%7C0%7C637953845107844869%7CUnknown%7CTWFpbGZsb3d8eyJWIjoiMC4wLjAwMDAiLCJQIjoiV2luMzIiLCJBTiI6Ik1haWwiLCJXVCI6Mn0%3D%7C3000%7C%7C%7C&sdata=aq5rkYEkZSsmptca52gBH5zlsp3VLHlOFr7lpR1kkBw%3D&reserved=0)  Contribute to minesneves/Zanzibar_population_genetics development by creating an account on GitHub.  github.com |
| --- | --- |

7) Thank you for stating "All remaining miracidial samples on Whatman FTA cards and associated DNA extractions have been bio-banked within the Schistosomiasis Collection at the Natural History Museum (SCAN)". Please note that your Data Availability Statement is currently missing the DOI/accession number of each dataset OR a direct link to access each database. If your manuscript is accepted for publication, you will be asked to provide these details on a very short timeline. We therefore suggest that you provide this information now, though we will not hold up the peer review process if you are unable.

I have now provided the direct SCAN links to DOI/databased links for all the miracidial samples stored at SCAN:

<https://www.nhm.ac.uk/our-science/our-work/sustainability/schistosomiasis-collection.html>

<https://scan.myspecies.info/>

**Responses to Reviewers**

**Reviewer #1: General comment**
*The experiment reported in the paper is in my opinion well conducted and the paper is very well written*

Thank you

*I think the main aim of the study: to assess the impact of the 5 years ZEST intervention  on the genetic structure of S. haematobium was probably not achievable, in the sense that schistosomes in Pemba were exposed to a very intense drug administration for several years and therefore expecting a measurable genetic change (in very similar arms) due to an intervention that lasted 5 years i was a little bit optimistic. However the study provide important information on other aspects of schistosomiasis control and therefore merits publications.*

Thank you, and indeed we fully agree – and have further emphasised this within our revised text, together with providing additional information of the past treatment histories of both islands.

*I have only minor suggestions:

1- in my opinion in material and methods section the authors should report  with a little more details the previous story of treatment with praziquantel in Pemba (that this is probably over 20 years longer). This is important because will put into prospective the activities conducted during ZEST.*

Agreed, as above, and now included.

*2. line 306 intensity of infection I think the author in addition to the main intensity of infection should also provide the intensity of infection by class (in order to give an idea of the changes occurred in the prevalence on these classes during ZEST).*

Thank you for this valid suggestion.

For urogenital schistosomiasis, infection intensities are classified into high where ≥50 e/10 ml and low where 1–49 e/10 ml (WHO 1998). The prevalence of heavy intensity infections was significantly higher on Pemba compared to Unjuga across all age-groups and time points (z= 5.63, *p*<0.0001). A significantly higher prevalence of heavy intensity infections was also found in adults compared to children of 6-8 years old, in Pemba (z= 3.484, p<0.05).

We have added this to our revised text.

*3-Figure 2 the unit of measure (eggs / 10 ml urine) of the mean intensity of infection should be indicated in the Y axis*

Agreed and amended.

Discussion

*4- I do not agree with the conclusion of the authors on line 438-39: “ difference in parasite fecundity also appeared to explain some of the hotspot observed” in my opinion from Figure 6ab shows that all range of parasite fecundity at baseline or follow can be present in  hot spot (both  in “brown” and “black” hotspot)*

Ok, we have rephrased this point from our revised discussion.

*Conclusion
5- line 510-511 I is not clear to me on which basis the authors mention “with the possible exception of an enhanced impact of molluscicide combined with MDA” the authors suggest that molluscicide combined with MDA has impact on genetic structure? It does not seems the case from this study.. can the authors better explain?*

Apologies if we were not sufficiently clear here – this related to the finding we cited (lines 330-331 of original manuscript) where there was ‘a trend towards a reduction in [allelic] diversity only being present in the Unguja MDA+snail control arm between baseline and Year 5 (*t*=-1.88, *p*=0.07)’. We have further clarified this within our revised text and added additional figures to our supplementary information section..

*Reviewer #2: Penance and colleagues describe a large-scale population genetic analysis of Schistosoma haematobium from two islands off the coast of Zanzibar. The rationale for the study was to examine the genetic consequence of different control strategies tested under a cluster-randomised trail, comparing ~1500 miracidia collected before and another ~1500 miracidia collected 5 years after intervention. The results presented suggests little genetic differentiation overall, but some variation in fecundity between and within islands, and some hints of genetic structure potentially indicative of sub-species variation. 

The manuscript is well written and the methodology appropriate. I think there could be some greater emphasis on showing results and data for some of the key conclusions which we lacking in places. I have made some suggestions below that are worth considering and may help the reader. Overall, this study has implications for the way in which schistosomiasis is controlled and emphasises the utility of genetics as part of the decision-making process, and will be a good fit for PLoS NTD.

I look forward to seeing a revised version. 

Kind regards, 

Stephen Doyle
Wellcome Sanger Institute*

Thank you, and we agree that the suggestions of both reviewers for additional clarity in places have improved our text.


*General comment
-       A key result is stated in the abstract, results and discussion is that there is a trend toward reduced genetic diversity over time. However, apart from the statistical test reported in the result, there are no data that shows this either in the results or supplementary data. Given this is a population genetic analysis, at least a table with allelic richness and inbreeding coefficients could/should be shown to support these conclusions.*

Agreed and amended. Whilst these showed little evidence of differential selection pressures on population genetic diversity between island or arm over time, we did not originally include additional data on these measures within our original manuscript -but we have now included these within our revised Supplementary Information Section.

*Specific comments*

*Key words
-       Haematobium is spelled incorrectly.
-       Could be consistent with capitalistion.*

Amdended

*Line 180: multiplexed microsatellite PCR panels (Panel 1 and Panel 2) as described in [22] [23, 25, 26].
-       Personally, I find it frustrating when reactions are not described. Could some details on the reaction setup and cycling conditions be included, even if in the supplement?
-       The information in the supplement, ie some primer sequences, looks incomplete as it is. Could this be checked?*

Agreed and amended – full details of all reactions, including where published elsewhere, have now been provided within the revised Supplementary Information file and cross-referenced within the main text.

*Line 196: “Actual numbers….”
-       Could those numbers be described?*

We have clarified our text here and added precise numbers to both our text and supplementary information tables.

*Line 201: “Data analyses”
-       Could the code used throughout be deposited in a stable repository?* 

Agreed and amended – we have uploaded the code and representative anonymised datafiles into an additional GitHub repository.

*Lines 183-186: “…cherry picked….”
-       Seems like there might be some redundant information here (cherry-picked is described twice for what I think is the same thing). Worth another check.*

There are two stages of cherry picking within our protocol – PCR products for analyses in the former and then the DNA elutions for Panel 2 PCR. We have revised our text to clarify this, as well as providing full SOP methodological details within our revised Supplementary Information file.

*Line 344: “sibship reconstruction”
-       I was excited to see this used, but felt like there was a missed opportunity to explore / describe the actual sibships, and whether there was any difference in the degree of sibship relationships between treatment arms or over time. Could this at least be commented on?
-       I appreciate the analysis does go on to estimate fecundity, but this is an average. Your genetic data (I presume) gives you precise(?) measures.*

Thank you and again a valid suggestion. Here we used the inferred number of females (and number of miracidia) from an original sibship reconstruction, as described in Neves et al., 2019 within the main text. In addition, we have now included an additional figure within our Supplementary information that shows the mean inbreeding coefficient by treatment arm, island and year – which neatly highlights only a difference again within the Unjuga MDA+mollusciding intervention arm by year 5 (Figure S3).

*Line 438: “Differences in parasite fecundity also appeared to explain some of the hotspots observed across islands and arms (Figure 6a,b).”
-       This seems like a bit of a weak conclusion from the Figure. Could this be more explicitly tested?*

As stated above, we have now revised and/or removed this point, and the text surrounding it. Instead we acknowledge that whilst we observe geographical differences in fecundity, they appeared to be inconsistently associated to persistent hotspots - and thus we conclude that fecundity differences may be a potential contributor to (and characteristic of) the observed hotspots, it is unlikely to be a sole driver.

*Line 445: “Further evidence of potential strain variations amongst parasites by both island, and potentially hotspot within islands, was revealed by PCA analyses“
-       There could be a bit more discussion around what is driving this difference within islands, esp given the distribution of hotspots, ie what factors lead to a hotspot, and what might lead to strain differences. The discussion is predominantly focused on between islands, not within islands.*

Agreed and amended, we have now expanded our revised text in relation to both between and within island.

*-       Is it not a bit curious that the variation explained by between islands in the PCA is basically the same was within island hotspot vs non-hotspot? I would have expected a stronger signal between islands than subsets of populations within islands.*

We agree that the between-island PCA clearly illustrates differentiation, and also, whilst slightly less pronounced, such PCA analyses also shows the within-island by hotspot differentiations – both in complement to that observed our other measures. This supports our conclusions that a range of, often overlapping, longer term environmental and anthropogenic factors appear to be influencing the population structure of the parasites observed today, all of which may also override any short term impacts as may be imposed by the ZEST treatment arms. We have aimed to clarify this further within our revised text.

*Line 483: “hotspots were found to be clustered with other hotspots“
-       Really? Is there a way to objectively define this?
-       Cross checking the cluster of highest mean fecundity populations in Fig 6a vs Fig 7a shows they are spread all over Pemba.*

As detailed above, we have amended this statement and surrounding discussions within our revised text.

*Line 485: “corroborating a recent study of S. haematobium in Zanzibar“*
*-       Reference?*

As detailed above, we have deleted this statement and surrounding discussions within our revised text.

*Line 514: ‘Whole Genome Sequencing“
-       Doesn’t need capitalisation*

Amended

*Figure 1 
-       It is very difficult to take anything away from this figure. It is much more like a graphical abstract rather than provide anything informative. 
-       I’d suggest either removing it, or adding information, sample sizes, a decision tree to describe how these different aspects of the study relate to each other. 
-       Some data from Supplementary Tables 1 and 2 might be useful here.*

Agreed and amended – we have removed Figure 1 and added additional sample size numbers by year, island, intervention arm and age grouping into our supplementary information document.

*Figure 4.
-       Is this figure needed? Figures 5 and 6 seem to cover this pretty well.* 

Agreed and amended. Whilst we saw advantages to illustrating the clear differences in fecundity between islands in our original Figure 4, we agree that this is also apparent from the subsequent figures by treatment arm (original Figure 5) and shehia (original Figure 6), and thus we have deleted this from our revised manuscript.

*Figure 6
-       Should there be some form of error bars on these mean estimates?*

A valid suggestion – however, we did not include error bars here as, in part, inclusion made the figures so visually complex/messy, they were difficult to view, but also more importantly because we used a mixed effects model and hence it would be problematic to define an efficient method that incorporates uncertainty into to random effects parameters. We have clarified this within our revised legend.

*-       Alternative to error bars – could it be worth combining the two plots (a+b), and use a different shape for the year? Could place a line connecting the two datapoints per site. That way, the reduction in fecundity between time points would be obvious. 
-       The variance looks much lower, especially in the 5 y group, than the confidence intervals show in Figure 5. Error bars in Fig 6 might be helpful showing this.*

As for the point above. Also, again combining the figures (which we have indeed tried also) was not deemed appropriate here, as again would make the figures difficult to interpret – particularly since not all hotspots that appear in Y1 also appear in Y5, and that all shehias are ordered on the x-axis by fecundity.

*-       Kinyasini_(Pemba) – should this be labelled in this way? Also, I couldn’t find this population on the maps in Fig 7.*

Kinyasini name has been amended accordingly.

*Figure 7
-       It is not quite clear to me what is the rationale for using a different colour scheme between a+b and c+d. Clearly, there are big differences between yr 1 and yr 5, however, different colour schemes prevent them from being compared?
-       Is the spatial distribution within a time point more important than between time points?
-       Could scale bars be placed on the maps?*

The colour scheme used in both figures is the same, but the scales are different between different years (fecundity ranges from 6.5-62 in Y1 and 4-17 Y5). Whilst we could make the scale the same on both, the issue is that within year 5, where the fecundities are much lower, the points will all have all the same colour if we use the scale from Y1 – and thus we would be unable to visually distinguish shehias with different fecundities within the same island. We have clarified this within our revised legend.

We have now added Kinyasini to this figure (which, as above, was a typo related to the name of the shehia, for which we are very grateful to this referee for spotting).

We have indeed scales on our figures.

*Figure 8
-       It would be good to show the axis labels with % variance on them, rather than in the figure legend*

Agreed and amended

*-       “d=0.5” is shown in all of the plots, but it not described anywhere. Perhaps remove from the plots, add it to the legend (if important to do so), and explain what it is.*

Agreed and amended – removed from plot and 'Scale represented as Euclidean distance, d = 0.5' added to the legend.

*-       Please describe what the ellipse represents in the legend.*

Agreed and included.

*Data availability: “No - some restrictions will apply”
-       Could this be explained in more detail?*

We have updated to clarify full data availability, and removed reference to any potential restrictions since the central open access databases are all anonymised.
